# Structural Analysis of *Pf*Sec62-Autophagy Interacting Motifs (AIM) and *Pf*Atg8 Interactions for Its Implications in RecovER-phagy in *Plasmodium falciparum*

**DOI:** 10.3389/fbioe.2019.00240

**Published:** 2019-09-25

**Authors:** Ashalatha Sreshty Mamidi, Ananya Ray, Namita Surolia

**Affiliations:** ^1^Molecular Biophysics Unit, Indian Institute of Science, Bangalore, India; ^2^Division of Biological Sciences, Indian Institute of Petroleum and Energy, Visakhapatnam, India; ^3^Molecular Biology and Genetics Laboratory, Jawaharlal Nehru Centre for Advanced Scientific Research, Bangalore, India

**Keywords:** autophagy, recovER-phagy, *Pf*Sec62-*Pf*Atg8 interactions, AIM/LIR motifs, *Plasmodium falciparum*

## Abstract

Autophagy is a degradative pathway associated with many pathological and physiological processes crucial for cell survival. During ER stress, while selective autophagy occurs via ER-phagy, the re-establishment of physiologic ER homeostasis upon resolution of a transient ER stress is mediated by recovER-phagy. Recent studies demonstrated that recovER-phagy is governed via association of Sec62 as an ER-resident autophagy receptor through its autophagy interacting motifs (AIM)/LC3-interacting region (LIR) toAtg8/LC3. Atg8 is an autophagy protein, which is central to autophagosome formation and maturation. *Plasmodium falciparum* Atg8 (*Pf*Atg8) has both autophagic and non-autophagic functions critical for parasite survival. Since *Plasmodium* also has Sec62 in the ER membrane and is prone to ER stress due to drastic transformation during their complex intraerythrocytic cycle; hence, we initiated the studies to check whether recovER-phagy occurs in the parasite. To achieve this, a comprehensive study based on the computational approaches was carried out. This study embarks upon identification of AIM sequences in *Pf*Sec62 by carrying out peptide-protein docking simulations and comparing the interactions of these AIMs with *Pf*Atg8, based on the molecular dynamic simulations. Detailed analysis is based on electrostatic surface complementarity, peptide-protein interaction strength, mapping of non-covalent bond interactions and rupture force calculated from steered MD simulations. Potential mean forces and unbinding free energies (ΔG_dissociation_) using Jarzynski's equality were also computed for the AIM/LIR motif complexes with *Pf*Atg8/*Hs*LC3 autophagy proteins to understand their dissociation free energy profiles and thereby their binding affinities and stability of the peptide-protein complexes. Through this study, we predict Sec62 mediated recovER-phagy in *Plasmodium falciparum*, which might open new avenues to explore novel drug targets for antimalarial drug discovery.

## Introduction

Autophagy is a catabolic process in which lysosomal hydrolases clear damaged sub cellular organelles and cytoplasmic constituents. Cells utilize this process to facilitate cellular homeostasis and viability during starvation and other stress conditions (Kroemer et al., 2010; Mizushima and Levine, 2010; Rubinsztein et al., 2012; Murrow and Debnath, 2013; Russell et al., 2014; Fuchs and Steller, 2015). Three principal types of autophagy are currently being recognized, viz., macroautophagy, microautophagy and in mammals, chaperone-mediated autophagy (Klionsky, 2004). While the characteristics of macroautophagy is generation of double membrane vesicles the autophagosomes, which subsequently fuse with lysosomes and release their contents into the lytic compartment, as part of single membrane autophagic bodies, the major feature of microautophagy is that the cytoplasmic constituents are taken up into lysosomes by invagination and inward budding of the lysosomal membrane. In the yeast and mammals, microautophagy can occur without a functional core autophagy machinery (Sattler and Mayer, 2000; Sahu et al., 2011). In chaperone mediated autophagy (CMA), targeted proteins are transported across the lysosomal membrane in a complex with chaperone proteins like Hsc-70 that are recognized the lysosomal membrane receptor, the lysosomal associated membrane protein 2A (LAMP-2A) resulting in their unfolding and degradation (Axe et al., 2008). In addition to macroautophagy/non-selective autophagy, which is generally induced by starvation, several autophagic processes selectively target particular organelles to lysosomes, such as peroxisomes, parts of nucleus, lipid droplets and endoplasmic reticulum and this is termed as selective autophagy (Stolz et al., 2014). Both selective and chaperone mediated autophagy mediates the delivery of cytoplasmic cargo via autophagosomes to the vacuole for degradation. This specific interaction is mediated by receptor proteins that link the cargo to the phagophore (the precursor of the autophagosome) membrane via the simultaneous interactions of receptor proteins with the cargo and the Atg8 family of proteins on the membrane. A number of Atg8/LC3 interacting motifs have been identified in a number of proteins, which bind to Atg8 protein in yeast and LC3 proteins in mammals. These short peptide motifs called Atg8-interacting motifs/LC3 interacting regions (AIM in yeast/LIRs in humans) are represented by a short consensus sequence [W/F/Y]xx[L/I/V], where “x” can be any amino acid and the core motif can be flanked at their N or C terminus by Ser, Thr and/or an acidic amino acid such as Glu and/or Asp (Birgisdottir et al., 2013; Hurley and Schulman, 2014; Rogov et al., 2014).

The selective autophagy mechanism might reflect the need to control the size of the organelles to eliminate damaged organelles or sub-domains of the organelles containing toxic materials. Based on the cargo delivered to the lysosomal compartment for clearance, these processes have been named aggrephagy for cytosolic protein aggregrates, ER-phagy for endoplasmic reticulum, mitophagy for mitochondria, pexoyphagy for peroxisomes, ribophagy for ribosomes and xenophagy for intracellular pathogens (Samson, 1981; Øverbye et al., 2007; Kraft et al., 2008; Till et al., 2012; Ashrafi and Schwarz, 2013; Deegan et al., 2013).

The endoplasmic reticulum (ER) is the site of protein synthesis in eukaryotic cells and is linked to autophagy in many ways. First, accumulation of misfolded proteins causes perturbation of ER homeostasis and induce stress response pathway termed as Unfolded Protein Response (UPR). Second, ER contributes to the autophagosome membrane (Axe et al., 2008; Hayashi-Nishino et al., 2009; Graef et al., 2013) and third, ER can also be a cargo for autophagy. The UPR increases production of ER-resident chaperones to reduce the load of misfolded proteins. Inability to restore ER functions can lead to activation of cell death or adaptation (Walter and Ron, 2011), hence, resolution of ER stress has to be followed by a recovery phase for re-establishing ER homeostasis. The recovery phase includes return of ER chaperones to the pre-stress levels, and is termed as recovER-phagy (Fumagalli et al., 2016). Recently, Sec62 a component of translocon complex has been identified as an ER-resident autophagy receptor involved in recovER-phagy (Fumagalli et al., 2016).

Sec62 selectively delivers excessive ER components to auto-lysosomal system for clearance during recovery from ER stress. Sec62 contains a conserved LC3 interacting region (LIR; in mammals) also known as Atg8 interacting motif (AIM; in yeast) in the C-terminal cytosolic domain which is needed for its function in recovER-phagy, but is dispensable for its function in protein translocon machinery. Sec62 thus has been identified as an important component in recovery of ER homeostasis.

*Plasmodium* has a limited set of autophagy proteins, and in general the role and functions of various autophagy proteins in Apicomplexans like *Plasmodium* sp. and *Toxoplasma gondii* is not fully understood (Latré de Laté et al., 2017). *Plasmodium falciparum* causes the most severe form of malaria, the cerebral malaria in humans. As the parasite has become resistant to the front line antimalarials such as chloroquine and artemisinin, there is an urgent need to identify newer targets for developing therapeutics. Recent findings of artemisinin resistance associated mutations in Atg18 (*Pf* Atg18) (Wang et al., 2016) and also alterations in *Pf* Atg8 distribution in chloroquine resistance (Gaviria et al., 2013) has created renewed interest in understanding autophagy like pathway and its various roles in this parasite. Among the autophagy related proteins, studies have been carried out mostly on *Pf* Atg8. Several findings have reported its multifunctional roles including apicoplast biogenesis and autophagy (van Dooren et al., 2000; Cervantes et al., 2014). *Pf* Atg8 is localized on apicoplast membrane as well as autophagosomes and is found to be lipidated, thus serving as the classical autophagy marker. Recently, our group reported that *Pf* Atg8 can be modulated by inhibitors and that starvation induced autophagy mediates parasite survival (Joy et al., 2018).

Findings of Fumagalli et al. (2016) have highlighted that during recovER-phagy the ER translocon component Sec62 interacts with LC3 protein, and this interaction is through LC3-interacting region (LIR). As *Plasmodium* has the translocon component *Pf* Sec62 and is constantly under stress condition due to its enormous growth and development during intra erythrocytic cycle (IEC), we initiated this study to find whether recovER-phagy occurs in this parasite through *Pf* Atg8-*Pf* Sec62 interactions, as to our knowledge no study has been reported recovER-phagy in any of the apicomplexans. Further, as the understanding of the structural basis of this phenomenon is essential; we dissected the Sec62-AIM motif interactions with the Atg8 protein. This analysis might open new avenues to propose novel targets for antimalarial drug development.

## Methodology

In this study, AIM sequences of *Pf* Sec62 capable of establishing specific contacts with the *Pf* Atg8 were identified through peptide-protein interactions. For this, we employed the *Pf* Atg3 AIM motif, NDWLLP interactions with *Pf* Atg8 and *Hs*Sec62, the LIR motif of humans—NDFEMI interactions with *Hs*LC3 as reference peptide-protein complexes.

### Molecular System Preparation of Atg8/LC3 Proteins

The three-dimensional coordinates of *P. falciparum* Atg8 (PDB ID: 4EOY; *Pf* Atg8) (Hain et al., 2012) and human LC3 (PDB ID: 4ZDV; *Hs*LC3) (Khaminets et al., 2015) were obtained from RCSB Protein Data Bank (https://www.rcsb.org). Pre-processing of protein coordinates was done by examining the assigned bond orders, adding missing atoms and hydrogens, filling missing loops and side-chains, checking the stereochemistry and atom's occupancy and deleting other hetero groups if present.

### Identification of *Pf*Sec62 AIM Sequences

AIM sequences in *P. falciparum* Sec62 (*Pf* Sec62) were identified using the mature protein sequence obtained from Uniprot (ID: Q8ILP6) (www.uniprot.org). The *Pf* Sec62 protein sequence was submitted to the iLIR web server (https://ilir.warwick.ac.uk) for the prediction of AIM peptides (Kalvari et al., 2014). The selected motifs of *Pf* Sec62, *Pf* Atg3 (NDWLLP) and *Hs*Sec62 (NDFEMI) were used in docking simulations.

### Peptide-Protein Docking Simulations

Following the preparation of molecular systems of Atg8/LC3 proteins, docking simulations with *Pf* Atg8 and the identified peptide motifs of *Pf* Sec62 were carried out. Dockings were performed between *Pf* Atg8 and *Pf* Atg3 NDWLLP motif as well as between *Hs*LC3 and *Hs*Sec62 NDFEMI. These two interactions served as references. After submission of a protein structure and peptide sequence, GalaxyPepDock server (http://galaxy.seoklab.org/pepdock) generated high-resolution complex structures based on protein structure and peptide-protein interaction similarities using the templates from PepBind database (Das et al., 2013). The three-dimensional models of peptide-protein complexes were generated using GalaxyTBM and refined by energy-based optimization of GalaxyRefine method (Lee et al., 2015). The predicted top 10 models were then scored using FireDock (Mashiach et al., 2008). The best model among the ten was further subjected to structural refinement using FlexPepDock (London et al., 2011). The best among the conformations was used to rank the binding affinities of AIM/LIR motifs to Atg8/LC3 proteins.

### Molecular Dynamic Simulations

The best docked conformation from each of the six AIM/LIR motif -Atg8/LC3 complexes, viz. *Pf* Sec62 QSYIDI—*Pf* Atg8, *Pf* Sec62 QSYIDI—*Pf* Atg8, *Pf* Sec62 SMYKSI—*Pf* Atg8, *Pf* Sec62 ENYDCL—*Pf* Atg8, *Pf* Sec62 TSFEEL—*Pf* Atg8, and *Hs*Sec62 NDFEMI—*Hs*LC3 were subjected to all-atom molecular dynamic simulations using GROMACS 5.1.1 (Abraham et al., 2015). Among which, the latter two peptide-protein complexes served as references to compare with the former four complexes during analyses. Molecular systems were prepared using OPLS-AA force field and TIP3P water model (Jorgensen et al., 1983; Robertson et al., 2015). Initially, energy minimization was performed *in vacuum* applying steepest descent algorithm for 1,000 steps and conjugate gradient minimization for 500 steps. Subsequently, periodic boundary conditions were defined by adjusting the boundaries of the cubic box by 10Å. Water and sodium ions were added to the unit cell to maintain overall charge neutrality and followed by energy minimization for 5,000 steps till the maximum force applied on the systems is <1,000 kJ/mol/nm. Position restrained MD simulations were performed to acclimatize the water molecules around the system in the unit cell followed by unrestrained simulations to equilibrate the solvated system. The molecular system was coupled to Berendesen thermostat set to 300 K and Parinello–Rahman barostat set to 1 atm pressure. All bonds were constrained using LINCS algorithm. Electrostatic calculations were accounted by particle-mesh ewald (PME) method with a cut-off distance for Coulomb and van der Waals interactions were maintained at 1.4 nm. The final production simulations for each peptide-protein complex was carried out for 50 ns.

### Preliminary Analysis of MD Trajectories

Preliminary analysis of molecular trajectories were performed by computing root mean square deviation (RMSD) on the backbone atoms of peptide-protein complexes. Dihedral principal component analysis (dPCA) was employed on the backbone coordinates of peptide-protein complexes to obtain stable conformations (Altis et al., 2007). Hydrogen bonds between the peptide-protein complexes were computed using gmx hbond module of gromacs. The components of non-covalent binding energy were calculated using the energy decomposition scheme of mmPBSA tool (Kumari et al., 2014).

### Computing Electrostatic Complementarity

The electrostatic potential surface was calculated around the AIM/LIR—Atg8/LC3 complexes using Adaptive Poisson–Boltzmann Solver (APBS) software (Jurrus et al., 2018). Initially, PDB2PQR webserver was employed for assigning charges and atomic radii based on the AMBER force field (Dolinsky et al., 2007). A fine grid spacing of 0.25 Å was used. Solvent effects were accounted for using a 0.15 M 1:1 electrolyte and dielectric constants of 2.0 and 78.54 for the protein interior and the solvent water, respectively. APBS software computes the potential surface by solving the non-linear PB equation in a grid-based approach.

### Computing Interaction Strength

The three dimensional atomic coordinates of the peptide-protein complexes were used for constructing protein structure graphs. Atoms and their non-covalent interactions in peptide-protein complexes were represented as nodes and edges, respectively. The atom pair contacts between the *Pf* Atg8/*Hs*LC3 and the peptide motifs were computed for the dPCA derived MD trajectories, which were then used to calculate the interaction strength. The interaction strength between two amino acid side-chains is calculated as a percentage following the method of Brinda and Vishveshwara (2005), the formula given as:

$$\begin{array}{l} I_{\text{ij}}=\frac{n_{\text{ij}}}{\sqrt{N_{i}\times N_{j}\times 100}} \end{array}$$

Where, n_ij_ is the number of atom pairs between the side chains of amino acid residues i and j, which are within a cut-off distance of 4.5 Å and N_i_ and N_j_ are the normalization factors of each residue types under interaction.

### Residue-Residue Based Non-covalent Bond Interaction Network

Ten molecular conformations of AIM/LIR peptide motifs and *Pf* Atg8/*Hs*LC3 proteins were retrieved intermittently from the dPCA derived trajectory and used to compute the amino acid based non-covalent bond interaction network. For this analysis, PPCheck webserver (http://caps.ncbs.res.in/ppcheck/) was used to construct the residue-residue networks and study the interaction energies based on non-covalent interactions.

### Steered MD Simulations

Constant-velocity steered MD (SMD) simulations were performed employing COM pulling method of Gromacs on the peptide-protein conformations obtained from the dPCA derived MD trajectory. The center of mass (COM) of the bound peptides (AIM/LIR motifs) were attached to an elastic spring and pulled from the binding site of *Pf* Atg8/*Hs*LC3 with a force constant set to 1,000 kJ/mol/nm along the pulling direction at two different rates, i.e., 0.010 nm/ns and 0.025 nm/ns. The SMD simulation ran until the peptide was completely pulled out of the binding site and entered the solvent region. The time length of SMD simulation was set to 5 ns to ensure complete dissociation of AIM/LIR peptides from the binding site of *Pf* Atg8/*Hs*LC3 proteins. The trajectories were saved for every 1 ps and also the steering forces. Ten SMD simulations were executed for each of the six peptide-protein complexes at two different velocities. Thus, a total of 120 SMD simulations were computed using different conformations extracted at various time points. Subsequently, rupture force and potential mean force (PMF) were calculated. The molecular trajectory was parsed to compute the center-of-mass (COM) distance for each of the complexes and the distance between the peptide-protein was re-computed at each time step to set to zero at the starting point.

### Potential Mean Force Calculations

Cumulant expansion of Jarzynski's equality was employed to compute the potential of mean force (PMF) from SMD simulations (Jarzynski, 1997). PMF represents the free energy change along the reaction coordinate. For this study, we calculated PMF profile using 10 SMD trajectories of each molecular complexes using the second order cumulant expansion of Jarzynski's equality between work and change in free energy, given as:

(1) $$\begin{array}{l} \Delta G=\langle W\;\rangle -\frac{\beta }{2}\left[\langle W^{2}\rangle -{\langle W\rangle }^{2}\right] \end{array}$$

where, W is the work done, β is equal to kT, and k is Boltzmann factor at a temperature T and ΔG is the free energy difference. The external non-equilibrium work done by the pulling force is obtained using the following:

(2) $$W=-kv\int_{0}^{t}\text{d}t^{\prime }\left[x(t^{\prime })-x_{0}-vt^{\prime }\right]$$

where k is the force constant for pulling and v is the pulling velocity. x(t′) and x_0_ are the reaction coordinate at t′ in the simulation and the relative initial position of the center of mass of the pulled peptide, respectively. The associated force f is calculated using:

(3) $$\begin{array}{l} f=-k\left[x\left(t^{\prime }\right)-x_{0}-vt^{\prime }\right] \end{array}$$

### Computation of Dissociation Constant (K_d_)

The change in free energy (ΔG_dissociation_) due to unbinding of Sec62 peptides from the Atg8/LC3 proteins computed using the second order cumulant expansion of Jarzynski's equality was used to calculate the dissociation constant (K_d_) based on the relationship between ΔG and K_d_.

(4) $$\begin{array}{l} \text{Δ}G=-\text{RT ln }{\text{K}}_{\text{d}} \end{array}$$

(5) $$\begin{array}{l} {\text{K}}_{\text{d}}={\text{e}}^{-\text{ΔG}/\text{RT}} \end{array}$$

where, *R* is the ideal gas constant (0.008314 kJ/mol) and T is the temperature in Kelvin.

## Results and Discussion

Autophagy is an essential physiological process involving the recognition of Atg8/LC3 machinery by recruiting cargo receptors constituting Atg8-interacting motif (AIM) or LC3-interacting region (LIR) for proper autophagosome formation (Birgisdottir et al., 2013). Recent studies on various key players regulating ER-phagy highlighted the involvement of Sec62—a component of the translocon, during the recovery phase of the ER stress (Fumagalli et al., 2016). This study invoked our interest in investigating whether recovER-phagy occurs in *Plasmodium falciparum* as this parasite is constantly under stress as it endures several modifications during its life cycle in two hosts. In view of the increasing interest in elucidating the AIM/LIR dependent regulation of autophagy pathway, understanding the role of Sec62 mediated selective autophagy during ER stress will open new avenues in developing highly effective antimalarial drugs inhibiting *Pf* Atg8-*Pf* Sec62 interactions.

### Identification of AIM/LIR Motifs in *Pf*Sec62

**Figure 1:**
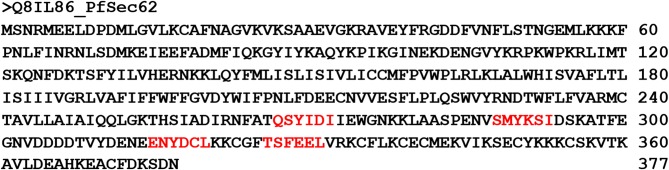
Protein **s**equence of *Pf*Sec62 highlighting the four AIM motifs in red, predicted using iLIR web server.

AIM/LIRs are short peptide motifs that mediate the binding of Atg8/LC3 to selective cargo receptor proteins. Hence, identification of specific AIM/LIR motifs of cargo receptor protein in *P. falciparum* is essential to understand the fundamental degradation processes and therein the functioning of selective autophagy machinery. Since, the structural information of Sec62 in *P. falciparum* (*Pf* Sec62) is unknown, we identified the AIM/LIR motifs from its protein sequence (Q8IL86) deposited in Uniprot database. *In silico* identification of functional AIM/LIRs predicted 13 hexa-peptide motifs, based on the simple match of [W/F/Y]xx[L/I/V] pattern in the protein sequence, of which four motifs at the C-terminal of *Pf* Sec62 viz. QSYIDI, SMYKSI, ENYDCL and TSFEEL were chosen based on PSSM score (Figure 1). Table S1 lists the four AIM/LIR motifs predicted in *Pf* Sec62 and considered for this study.

### Docking AIM/LIR Motifs to Atg8/LC3, the Core Autophagy Proteins

**Figure 2:**
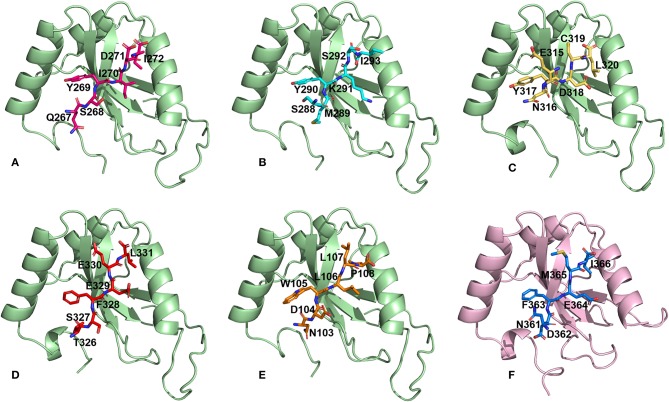
Docked poses of Sec62-AIM/LIR motifs in *Pf*Atg8/*Hs*LC3 autophagy proteins. Binding pose of **(A)** QSYIDI (magenta), **(B)** SMYKSI (cyan), **(C)** ENYDCL (yellow), **(D)** TSFEEL (red), and **(E)** NDWLLP (orange) with *Pf*Atg8 (light green), and **(F)** NDFEMI (blue) in *Hs*LC3 (pink).

We then performed docking simulations using the four identified AIM sequences of *Pf* Sec62: QSYIDI, SMYKSI, ENYDCL and TSFEEL. Docking calculations between the known AIM sequence of *Pf* Atg3 (NDWLLP) and *Pf* Atg8 and LIR sequence of *Hs*Sec62 (NDFEMI) and *Hs*LC3 served as references to identify the *Pf* Atg8 specific AIM sequences in *Pf* Sec62. To validate the accuracy of docking results, we superposed the docked complex of *Pf* Atg8- *Pf* Atg3 NDWLLP on the x-ray crystallography determined structure (PDB ID: 4EOY) to examine the positioning of *Pf* Atg3 NDWLLP. We observed that the peptide was exactly placed in the W and L sites of *Pf* Atg8, thus confirming the precision of docking calculations (Figure S1). Docking performed using GalaxyPepDock constrained the AIM/LIR peptides in the binding sites of Atg8/LC3 proteins based on the peptide-protein interface information of peptide-protein complexes deposited in the Protein Data Bank (PDB). It is already known that AIM/LIR motifs recognize and bind to the W- and L-sites of *Pf* Atg8 and *Hs*LC3 and hence we selected the complexes that positioned the peptides only at the binding site (Noda et al., 2010). For predicting high-resolution peptide-protein complexes, conformations were subjected to high-throughput refinement that allowed flexibility in backbone and side-chains of peptides and proteins and rescored. The global energy of the peptide-protein complex is used as score to rank the docked models. Hence, lower the score better is the binding interaction (Table S2). Subsequently, ten high-resolution conformations were generated, of which the best pose was selected for each AIM/LIR-Atg8/LC3 complexes. Among all the peptide-protein complexes, we noted that the binding affinity of *Pf* Sec62 SMYKSI for *Pf* Atg8 is worse (-51.45) as compared to other three peptides of *Pf* Sec62: QSYIDI (−81.84), ENYDCL (−77.81), and TSFEEL (−78.54). Energies of NDWLLP (*Pf* Atg3) and NDFEMI (*Hs*Sec62) when bound to *Pf* Atg8 and *Hs*LC3 autophagy proteins were observed to be −73.62 and −95.67, respectively. Thus, the best docked conformation selected from each AIM/LIR-Atg8/LC3 complexes were taken as initial conformations for MD simulations. Figure 2 presents the docked poses of various AIM/LIR peptides at Atg8/LC3 binding sites.

### Structural Refinement and Extraction of Stable Conformations

MD simulations shed light on the biomolecular processes at atomic level providing information about the conformational dynamics and binding interactions, which are sometimes not amenable through experimental methods. In this work, we performed classical MD simulations for the previously mentioned six AIM/LIR-Atg8/LC3 complexes for 50 ns. Structural changes induced during MD simulations were evident from the backbone fluctuations of peptide-protein complexes plotted as RMSDs computed as a function of time (Figure S2). Since, considering the entire MD trajectory for analysis will result in approximations, we employed dihedral PCA on the backbone atoms to accurately separate the internal and overall dynamics of the conformations (Altis et al., 2007). The conformations constituting well-separated minima correspond to specific conformational states of the free energy landscapes, which were merged to exclude the random conformations and to obtain stable structural sub-states for analysis (Mamidi and Surolia, 2015). Figure S2 presents the RMSD plots computed for the AIM/LIR-Atg8/LC3 complexes and the structurally stable conformations constituting the sub-states retrieved from dPCA. Table 1 details the total number of conformations obtained post-dPCA analysis for all the six peptide-protein complexes and their respective RMSDs computed over the entire 50 ns MD trajectory and for the post-dPCA derived MD trajectory. Here, we observed that the average RMSD of the dPCA derived MD trajectory of TSFEEL (*Pf* Sec62)—*Pf* Atg8 complex was high indicating that the complexation has resulted in considerable conformational and structural changes, whereas in *Pf* Atg8-SMYKSI, RMSD was low. A keen observation on the decrease in standard deviation between the RMSDs of whole trajectory and the post-dPCA trajectory attest the filtering of random conformations from the stable sub-states thus providing structurally similar and highly stable conformations (Table 1). Thus, we believe that studying the conformations constituting the structurally stable sub-states will shed light on the key differences among the AIM/LIR—Atg8/LC3 complexes during peptide-protein interaction analyses.

**Table 1:** Conformations obtained from structurally stable states and the RMSDs.

**AIM/LIR motifs**	**Autophagy protein**	**Conformations in structurally stable**	**RMSD**	
			**50 ns MD trajectory**	**Stable conformations**
*Pf*Sec62 QSYIDI	*Pf*Atg8	14888	0.24 ± 0.04	0.25 ± 0.03
*Pf*Sec62 SMYKSI		17821	0.17 ± 0.02	0.17 ± 0.02
*Pf*Sec62 ENYDCL		15590	0.21 ± 0.02	0.20 ± 0.02
*Pf*Sec62 TSFEEL		19705	0.26 ± 0.05	0.30 ± 0.02
*Pf*Atg3 NDWLLP		13568	0.25 ± 0.03	0.25 ± 0.02
*Hs*Sec62 NDFEMI	*Hs*LC3	11125	0.25 ± 0.03	0.27 ± 0.01

### Energy Profiles of Atg8/LC3-AIM/LIR Motif Complexes

**Figure 3:**
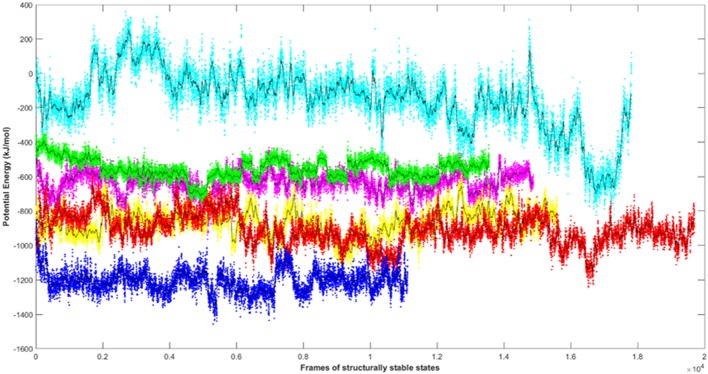
Plot showing the potential energy profiles of the six AIM/LIR—*Pf*Atg8/*Hs*LC3 complexes computed for the structurally stable conformations. The energies represented as line plots in different colors depicts *Pf*Sec62 QSYIDI (magenta), *Pf*Sec62 SMYKSI (cyan), *Pf*Sec62 ENYDCL (yellow), *Pf*Sec62 TSFEEL (red), *Pf*Atg3 NDWLLP (green) upon binding to *Pf*Atg8 and *Hs*Sec62 NDFEMI (blue) on interacting with *Hs*LC3. The line in black indicates the moving average calculated for the energies over 50 conformations.

Since the short-range van der Waals interactions and long-range electrostatic interactions determine the nature of the protein binding dynamics, we report the profiles of potential energies computed for the six peptide-protein complexes mentioned earlier. Figure 3 shows the total energy profiles of the AIM/LIR-Atg8/LC3 complexes computed for the post-dPCA derived MD trajectory. The average potential energy of the reference complexes: NDFEMI (*Hs*Sec62) - *Hs*LC3- and NDWLLP (*Pf* Sec62) - *Pf* Atg8 were −1211.33 ± 71.42 and −557.03 ± 62.82 kJ/mol, respectively. On the other hand, the four AIM peptides of *Pf* Sec62: QSYIDI, SMYKSI, ENYDCL, and TSFEEL upon interaction with *Pf* Atg8, exhibited potential energies of −643.33 ± 71.77, −177.94 ± 97.46, −852.89 ± 76.03 and −908.88 ± 90.58 kJ/mol, respectively. Table 2 provides the van der Waals and electrostatic energy components contributing to the potential energies of all the six peptide-protein complexes. We have noticed that the high binding affinity of NDFEMI (*Hs*Sec62) to *Hs*LC3 is due to favorable electrostatic energy. Similar observations were reported for ENYDCL and TSFEEL peptides of *Pf* Sec62, which exhibited relatively high binding affinity to *Pf* Atg8, followed by *Pf* Sec62 QSYIDI and *Pf* Atg3 NDWLLP. On the contrary, *Pf* Sec62 SMYKSI showed unfavorable electrostatic interactions. As negative potential corresponds to attraction and positive potential to repulsion, binding of *Pf* Sec62 TSFEEL to *Pf* Atg8 is believed to stabilize *Pf* Atg8 in comparison with other peptides especially *Pf* Sec62 SMYKSI, which destabilized the complex formation due to its unfavorable electrostatic potential energy.

**Table 2:** Non-covalent bond interaction energy of the AIM/LIR - *Pf*Atg8/*Hs*LC3 complexes.

**AIM/LIR motifs**	**Autophagy protein**	**Total**	
		**van der Waals Energy**	**Electrostatic Energy**
*Pf*Sec62 QSYIDI	*Pf*Atg8	−187.88 ± 18.59	−455.33 ± 71.26
*Pf*Sec62 SMYKSI		−203.37 ± 23.57	25.43 ± 97.43
*Pf*Sec62 ENYDCL		−152.53 ± 19.08	−700.35 ± 83.90
*Pf*Sec62 TSFEEL		−152.72 ± 23.06	−756.16 ± 93.64
*Pf*Atg3 NDWLLP		−194.04 ± 21.32	−368.98 ± 68.24
*Hs*Sec62 NDFEMI	*Hs*LC3	−162.04 ± 22.90	−1,049 ± 68.85

### Mapping of Electrostatic Potentials for Surface Complementarity

**Figure 4:**
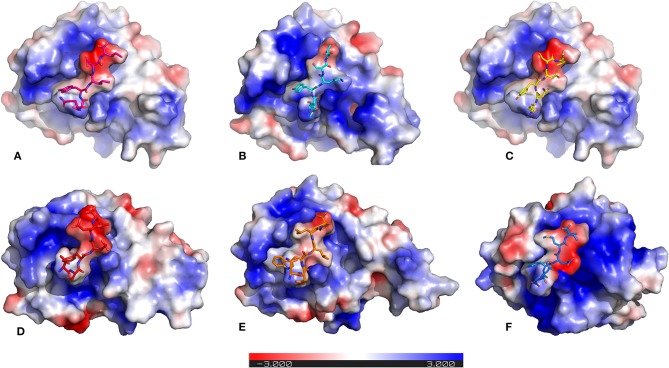
Electrostatic surface potentials mapped on the six AIM/LIR—*Pf*Atg8/*Hs*LC3 complexes reveal their charge complementarity. The binding position of peptides – **(A)** *Pf*Sec62 QSYIDI (magenta), **(B)** *Pf*Sec62 SMYKSI (cyan), **(C)** *Pf*Sec62 ENYDCL (yellow), **(D)** *Pf*Sec62 TSFEEL (red), and **(E)** *Pf*Atg3 NDWLLP (orange) in *Pf*Atg8 and **(F)** *Hs*Sec62 NDFEMI (blue) in *Hs*LC3 are also represented in sticks for better understanding. Blue surface on the protein represents positive charge and red represents negative charge and white neutral.

It is previously known that electrostatic surface potentials can modulate the binding behavior of peptide-protein complexes (McCoy et al., 1997; Sheinerman et al., 2000). Hence, we calculated the electrostatic fields for autophagy proteins and the peptide motifs to visualize and understand their electrostatic charge complementarity (Figure 4). We noticed that there was a significant electrostatic complementarity between NDFEMI (*Hs*SEc62)–*Hs*LC3, *Pf* Sec62 QSYIDI–*Pf* Atg8; *Pf* Sec62 ENYDCL–*Pf* Atg8, and *Pf* Sec62 TSFEEL–*Pf* Atg8 complexes, whereas *Pf* Atg3 NDWLLP–*Pf* Atg8 and *Pf* Sec62 SMYKSI–*Pf* Atg8 showed relatively low surface complementarity. According to the above findings, the *Pf* Sec62 TSFEEL–*Pf* Atg8 complex as in *Hs*Sec62 NDFEMI–*Hs*LC3 conferred stability due to electrostatic surface complementarity at the interface compared to other peptide motifs. On the other hand, *Pf* Sec62 SMYKSI–*Pf* Atg8 showed very low electrostatic surface complementarity among other motifs.

### Specific Non-covalent Interactions of AIM/LIR-Atg8/LC3 Complexes

**Figure 5:**
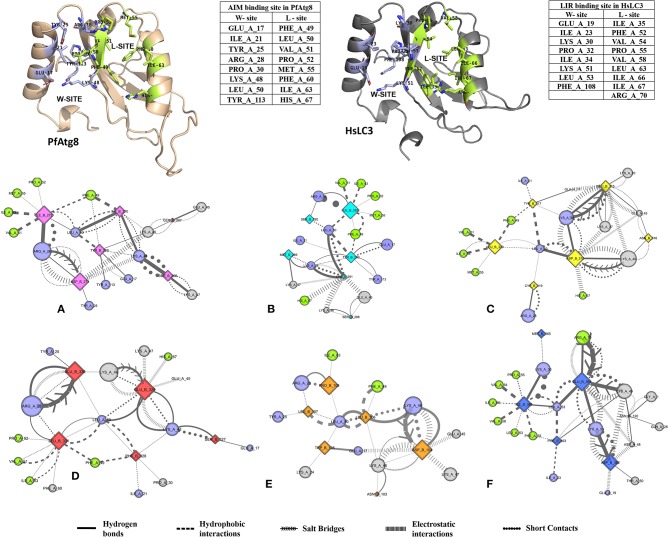
Non-covalent bond interactions in AIM/LIR - *Pf*Atg8/*Hs*LC3 complexes. **(A)** *Pf*Sec62 QSYIDI (magenta), **(B)** *Pf*Sec62 SMYKSI (cyan), **(C)** *Pf*Sec62 ENYDCL (yellow), **(D)** *Pf*Sec62 TSFEEL (red) and **(E)** *Pf*Atg3 NDWLLP (orange) LIR motifs bound to *Pf*Atg8 and **(F)** *Hs*Sec62 NDFEMI (blue) bound to HsLC3. The upper panels show W-site and L-site in *Pf*Atg8 and *Hs*LC3 in cartoons and adjacent tables detail the residues composing the respective binding sites. The non-covalent bond interactions between the AIM/LIR motifs and *Pf*Atg8/*Hs*LC3 are represented as network maps shown as different line types.

**Figure 6:**
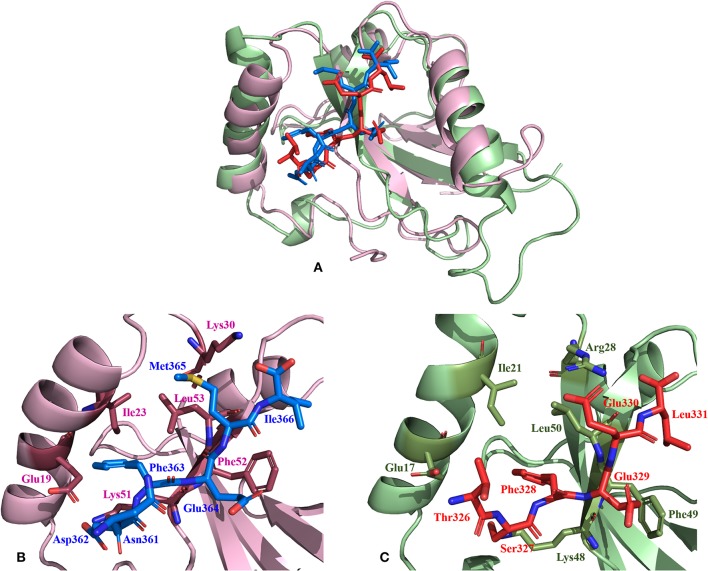
Relative comparison of the binding interactions of *Hs*Sec62 NDFEMI—*Hs*LC3 and *Pf*Sec62 TSFEEL—*Pf*Atg8 peptide-protein complexes. **(A)** Super-positioning of *Hs*Sec62 NDFEMI—*Hs*LC3 (shown in pink) and *Pf*Sec62 TSFEEL—*Pf*Atg8 (shown in green color) showing similarities in the peptide-protein complexes; **(B)** Binding interactions of *Hs*Sec62 NDFEMI with the residues of W-site of *Hs*LC3 and **(C)** binding interactions of *Pf*Sec62 TSFEEL with the residues of W-site of *Pf*Atg8.

Non-covalent interactions across the interfaces owe to van der Waals, hydrogen-bonding and hydrophobic contacts that render stability to the two interacting partners for an enhanced molecular recognition and structural specificity. Hence, identifying the relative differences in non-covalent bond interactions can characterize the binding strength between the appropriate cognate partners (Biswal et al., 2005). The strength of interactions between the Atg8/LC3 and the Sec62 AIM sequences were evaluated based on the physicochemical compatibility of residue types and their propensity to make maximum contacts during complex formation. We have observed that *Pf* Atg8 and *Hs*LC3 autophagy proteins were bound to *Pf* Atg3 NDWLLP and *Hs*Sec62 NDFEMI (with an interaction strength of 8.58 and 8.88, respectively. In the same way, interaction strength computed for the four *Pf* Sec62-peptides yielded a score of 2.89, 6.66, 7.48 and 8.23 for QSYIDI, SMYKSI, ENYDCL and TSFEEL, respectively. We initially studied the non-covalent bond interactions for the MD trajectory of *Pf* Atg3 NDWLLP - *Pf* Atg8 complex and compared with the interactions of the x-ray crystallography determined complex. As reported by Hain et al. (2012), we found that Asp103 of *Pf* Atg3 NDWLLP formed a salt-bridge with Lys46 of *Pf* Atg8 and the side chains of Trp105 and Pro108 of *Pf* Atg3 NDWLLP were deeply docked into the W and L-sites of *Pf* Atg8, respectively (Tables S3, S7). In addition, other residues of the peptide also established several non-covalent interactions at the binding site of *Pf* Atg8. Thus, we confirmed that docking and MD simulations reproduced experimental results and were confident to carry out further analysis for other AIM/LIR-Atg8/LC3 complexes. Figure 5 shows the network maps representing the non-covalent bond interactions between the AIM/LIR peptides and the Atg8/LC3 proteins. A keen observation of the network maps revealed that C-terminal residue (Pro108) of *Pf* Atg3 NDWLLP motif showed prominent interactions with both polar and non-polar amino acid residues in the L-site of *Pf* Atg8, whereas the C-terminal residue (Ile366) of NDFEMI, an LIR motif in *Hs*Sec62 is positioned in a strong hydrophobic field surrounded by non-polar residues of the L-site. Similar to *Hs*Sec62 NDFEMI - *Hs*LC3 complex, the C-terminal residues of the four *Pf* Sec62-peptide motifs were found to be fenced by the hydrophobic residues in the L-site of *Pf* Atg8. Additionally, ENYDCL and TSFEEL of *Pf* Sec62 formed salt-bridges with the same residues of the *Pf* Atg8 as in *Hs*Sec62 NDFEMI - *Hs*LC3 complex. Surprisingly, we observed that the non-bonded interactions found between *Hs*Sec62 NDFEMI and *Hs*LC3 were also conserved between *Pf* Sec62 TSFEEL and *Pf* Atg8 (Figure 5). Detailed examination revealed that the W-site of *Hs*LC3 exhibited non-covalent interactions with *Hs*Sec62 NDFEMI through the following contacts between residues – (i) Glu19 (*Hs*LC3) – Asp362 (second residue of NDFEMI), (ii) Ile23, Phe52 and Leu53 (HsLC3) – Phe363 (third residue of NDFEMI), (iii) Lys51 (*Hs*LC3) –Glu364 (fourth residue of NDFEMI), (iv) Lys30 (*Hs*LC3) – Met365 (fifth residue of NDFEMI) and (v) Leu53 (*Hs*LC3) – Ile366 (sixth residue of NDFEMI). Comparable network of non-bonded interactions was also observed with *Pf* Sec62 TSFEEL and the residues of the W-site in *Pf* Atg8. The interactions were noted between (i) Glu17 (*Pf* Atg8) – Ser327 (second residue of TSFEEL), (ii) Ile21, Phe49, Leu50 (*Pf* Atg8) - Phe328 (third residue of TSFEEL), (iii) Lys48 (*Pf* Atg8) – Glu329 (fourth residue of TSFEEL), (iv) Arg28 (*Pf* Atg8) – Glu330 (fifth residue of TSFEEL) and (v) Leu50 (*Pf* Atg8) – Leu331 (sixth residue of TSFEEL). Figure 6 highlights that the structural orientations of *Pf* Sec62 TSFEEL in the binding site of *Pf* Atg8 and *Hs*Sec62 NDFEMI in the binding site of *Hs*LC3 are highly similar. Our observations were further strengthened by the formation of hydrogen bonds, which are known to be directional and contribute to molecular specificity and recognition (see Table S3). Even, the residue-wise potential energies for the terminal residues of *Pf* Sec62 TSFEEL were found to be low indicating favorable interactions with the W and L-site of *Pf* Atg8 in comparison to other peptide motifs (Tables S4, S5). On the other hand, such conservation in peptide-protein binding interactions were not observed with the other three peptides (QSYIDI, SMYKSI and ENYDCL of *Pf* Sec62) in *Pf* Atg8. Thus, we report that *Pf* Sec62 TSFEEL qualifies as AIM/LIR motif showing non-covalent connections with *Pf* Atg8 in par with that of *Pf* Atg3 NDWLLP and *Hs*Sec62 NDFEMI with their respective Atg8/LC3 proteins.

### Mechanical Force of Disruption as a Measure of Binding Affinity

**Figure 7:**
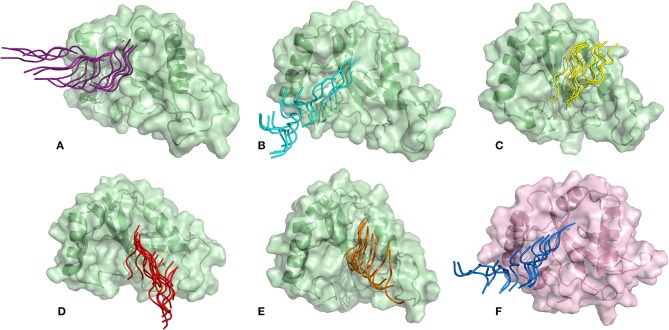
Dissociation of AIM/LIR peptides from the binding site of their respective Atg8/LC3 autophagy proteins: **(A)** *Pf*Sec62 QSYIDI (magenta), **(B)** *Pf*Sec62 SMYKSI (cyan), **(C)** *Pf*Sec62 ENYDCL (yellow), **(D)** *Pf*Sec62 TSFEEL (red), and **(E)** *Pf*Atg3 NDWLLP (orange) LIR motifs bound to *Pf*Atg8 (green), and **(F)** *Hs*Sec62 NDFEMI (blue) bound to HsLC3 (pink).

**Figure 8:**
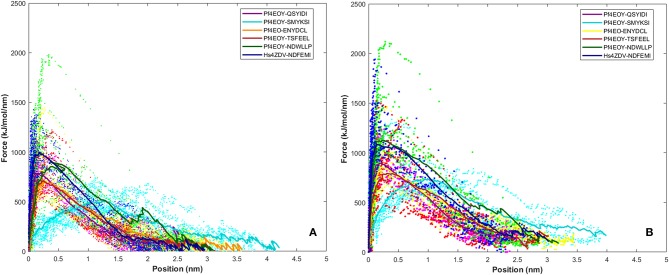
Rupture force profile computed for the AIM/LIR - *Pf*Atg8/*Hs*LC3 complexes at pull velocities **(A)** 0.010 nm/ns and **(B)** 0.025 nm/ns for the LIR motifs (i) *Pf*Sec62 QSYIDI (magenta), (ii) *Pf*Sec62 SMYKSI (cyan), (iii) *Pf*Sec62 ENYDCL (yellow), (iv) *Pf*Sec62 TSFEEL (red) and (v) *Pf*Atg3 NDWLLP (orange) LIR motifs bound to *Pf*Atg8 and (vi) *Hs*Sec62 NDFEMI (blue) bound to HsLC3. The dotted lines represent the individual dissociation profile of 10 SMD trajectories for each peptide-protein complex and the solid line represents their average.

SMD is a non-equilibrium simulations technique mimicking the principle of atomic force microscopy (AFM) to study the dissociation of biomolecular complexes and measure the transient and dynamical strength of receptor-ligand interactions (Evans et al., 1995; Deufhard et al., 1997). It is a stochastic process requiring pulling to be performed several times and averaged on multiple trajectories (Di Palma et al., 2015). Several SMD studies were successful in ranking the top leads as potential inhibitors based on their relative binding affinities (Mai et al., 2010; Mai and Li, 2011; Ngo et al., 2016). Here in this study, we implemented SMD to investigate the relative dissociation rates of various Atg8/LC3-peptide complexes and understand their unbinding regimes (Figure 7). Since the detachment rate determines the bond strength and the gradient of binding free energy, two sets of ten individual SMD simulations performed at different pull velocities (0.010 nm/ns and 0.025 nm/ns) were computed for each peptide-protein complex and the rupture force is presented as an average in Figure 8. The rupture force profiles of the two reference peptide motifs, i.e., *Pf* Atg3 NDWLLP with *Pf* Atg8 and *Hs*Sec62 NDFEMI with *Hs*LC3 when pulled with different velocities of 0.010 nm/ns and 0.025 nm/ns were found to be similar and relatively high compared to the four peptide motifs of *Pf* Sec62. The rupture force of dissociation for *Pf* Atg3 NDWLLP from the binding site of *Pf* Atg8 was found to be 905.88 kJ/mol/nm at 0.36 nm and 1127.71 kJ/mol/nm at 0.24 nm, when pulled at a constant velocity of 0.010 nm/ns and 0.025 nm/ns, respectively. Similarly, HsSec62 NDFEMI also dissociated from *Hs*LC3 with a rupture force noted at a pull force of 1000.96 kJ/mol/nm and pull distance of 0.16 nm at a constant velocity of 0.010 nm/ns and a pull force of 1084.82 kJ/mol/nm and pull distance of 0.14 nm at a constant velocity of 0.025 nm/ns. We noted that the force required for dissociating the three AIM motifs of *Pf* Sec62, i.e., QSYIDI, ENYDCL and TSFEEL was high and showed similar rupture profiles at both the pull velocities and dissociated at a pull distance <0.5 nm from the binding site. On the other hand, *Pf* Sec62 SMYKSI showed least rupture force of dissociation from *Pf* Atg8. Furthermore, we computed the distances between the center of masses (COM) of peptides and proteins in the complexes and noticed that *Pf* Sec62 SMYKSI was bound to the binding site for a longer time in comparison with the other three peptides of *Pf* Sec62 and reference peptides (Figure S3). The above findings highlight that *Pf* Sec62 SMYKSI might have been involved in other non-specific interactions as the dissociation profile did not exhibit significant drop in the pull force even when the motif was pulled away from the binding site of *Pf* Atg8.

### Potential Mean Force Profile Confers the Relative Binding Affinities Between AIM/LIR-Atg8/LC3 Complexes

**Figure 9:**
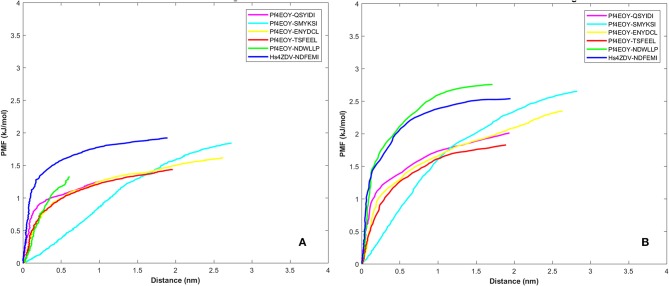
Potential mean force profiles computed and plotted for the AIM/LIR—*Pf*Atg8/*Hs*LC3 complexes at pull velocities **(A)** 0.010 nm/ns and **(B)** 0.025 nm/ns for the LIR motifs (i) *Pf*Sec62 QSYIDI (magenta), (ii) *Pf*Sec62 SMYKSI (cyan), (iii) *Pf*Sec62 ENYDCL (yellow), (iv) *Pf*Sec62 TSFEEL (red) and (v) *Pf*Atg3 NDWLLP (orange) LIR motifs bound to *Pf*Atg8 and (vi) *Hs*Sec62 NDFEMI (blue) bound to HsLC3.

Dissociation free energy along a reaction coordinate provides a glimpse on the strength of non-covalent bond interactions between the intermolecular complexes. The free energy changes of the dissociation were extracted from potential mean force (PMF) using the Jarzynski's equality computed based on the second-order cumulant expansion (Vashisth and Abrams, 2008). This proved advantageous in avoiding the overestimation of free energy changes by averaging over multiple trajectories and to correct the unphysical trajectories. Previously, Jarzynski's equality for PMF was used to study the self-assembly of peptides (Yu et al., 2013), peptide designing strategy (Lesitha et al., 2017), protein-protein interactions (Cuendet and Michielin, 2008) and in ranking the peptide of the docked complexes (Ngo et al., 2016). Considering the above studies and suitability of Jarzynski's equality to compute the PMF using low number of trajectories from SMD, we resorted to apply this post-processing method to estimate the work done during the dissociation of the four peptides of *Pf* Sec62 from the binding site of *Pf* Atg8 (Park et al., 2003; Park and Schulten, 2004). The potential mean force profiles at the two constant velocities−0.010 nm/ns and 0.025 nm/ns indicated that the PMF curve for *Pf* Atg3 NDWLLP and *Hs*Sec62 NDFEMI bound to *Pf* Atg8 an *Hs*LC3, respectively, were nearly similar and relatively high compared to the other four *Pf* Sec62 motifs. The PMF plots in Figure 9 represent the dissociation PMF profiles of the three peptides of *Pf* Sec62– QSYIDI, ENYDCL and TSFEEL, which showed a similar trend of increase in force to disrupt the binding interactions with *Pf* Atg8, whereas *Pf* Sec62 SMYSKI showed an altogether different PMF profile with low force gradient required during the dissociation process. A net positive free energy change between the initial and end states demonstrate stability of bound complex at equilibrium (Vashisth and Abrams, 2008). We observed that the difference in free energy of *Pf* Atg3 NDWLLP peptide from *Pf* Atg8 and *Hs*Sec62 NDFEMI from *Hs*LC3 was noted to be 1.33 and 1.91 kJ/mol at a constant velocity of 0.010 nm/ns and 2.75 and 2.53 kJ/mol at a velocity of 0.025 nm/ns, respectively. Similar calculations for the three *Pf* Sec62 motifs—QSYIDI, ENYDCL and TSFEEL pulled from *Pf* Atg8 were found to be 1.25, 1.61, and 1.43 kJ/mol at a constant velocity of 0.010 nm/ns and 2.01, 2.34, and 1.82 kJ/mol pulled at a constant velocity of 0.025 nm/ns, respectively. On the other hand, although *Pf* Sec62 SMYKSI exhibited relatively high free energy change recorded as 1.84 kJ/mol at a constant velocity of 0.010 nm/ns and 2.65 kJ/mol at a velocity of 0.025 nm/ns, the dissociation profile shows that it reaches equilibrium much slower than the other *Pf* Sec62 motifs. In short, the work done on the former three peptides of *Pf* Sec62 and the two reference peptides interactions is high and allows the transition to occur near the equilibrium, unlike with *Pf* Sec62 SMYKSI. This implies that *Pf* Sec62 SMYKSI has poor and non-specific binding with *Pf* Atg8, which is in accord with the low electrostatic complementarity as discussed earlier. Figure 9 shows the PMF profiles of the AIM/LIR peptides bound to *Pf* Atg8/*Hs*LC3 estimated at two velocities−0.010 nm/ns and 0.025 nm/ns.

### Free Energies Changes Due to Dissociation/Association of Sec62-Atg8/LC3 Complexes

To check whether the free energies of dissociation will add more values to the rupture force and PMF profile, we computed the change in free energies (ΔG) and dissociation constant (K_d_) based on multiple steered MD trajectories for the six Sec62–Atg8/LC3 complexes performed at constant pull velocities of 0.010 nm/ns and 0.025 nm/ns (Table S6). We observed that the change in free energies of dissociation are high at low pull velocity (0.010 nm/ns) than at 0.025 nm/ns. *Pf* Atg3 NDWLLP peptide bound to *Pf* Atg8 and *Hs*Sec62 NDFEMI bound to *Hs*LC3 showed high ΔG and low K_d_ in comparison with other *Pf* Sec62-*Pf* Atg8 complexes indicating relatively higher binding affinity. Among the four *Pf* Sec62 peptides: QSYIDI and TSFEEL exhibited high ΔG and low K_d_ implying better binding with *Pf* Atg8 relative to SMYKSI and ENYDCL based on the constant velocity SMD performed at 0.010 nm/ns. On the contrary, reverse is observed between the four *Pf* Sec62 peptides from SMD simulations at 0.025 nm/ns. Although, ΔG_dissociation_ serves as an estimate for analyzing relative binding affinities, we did not observe any significant trend in the change in free energies at both the pull velocities to rank the peptides. Hence, we even compared the ΔG_binding_ for the six peptide-protein complexes calculated using mmPBSA method and noted that *Pf* SMYKSI exhibited low binding affinity, whereas *Pf* TSFEEL exhibited relatively high binding affinity. Due to this ambiguity and mixed trend observed in the ΔG_dissociation_ and ΔG_binding_, these did not prove advantageous in ranking the *Pf* Sec62 peptides but analyzing the rupture force and PMF profiles helped identify the relative strength of peptide binding interactions with *Pf* Atg8.

## Conclusions

This work is a comprehensive study toward identifying the interactions between AIM/LIR motifs of *Pf* Sec62 and *Pf* Atg8 to decipher existence of Sec62 mediated recovER-phagy in *P. falciparum*. Four peptide AIM sequences of *Pf* Sec62 were chosen from *Pf* Sec62 C-terminal and subjected to similarity-based peptide-protein docking calculations followed by energy optimization. The best binding modes of these peptides to *Pf* Atg8/*Hs*LC3 complexes were computed. MD simulations captured the functional dynamics of the selected peptides with *Pf* Atg8/*Hs*LC3 complexes based on the dPCA and derived ensemble of metastable conformations to carry out further analysis. Our studies indicate that the *Hs*Sec62 motif bound to *Hs*LC3with high affinity as compared to the binding of *Pf* Atg3 NDWLLP to *Pf* Atg8. *Pf* Atg8-*Pf* Atg3 interactions are already well studied. Since we presumed that, the *Pf* Sec62 motif should interact with *Pf* Atg8 with higher binding affinity than the other decoy motifs and we studied the various aspects of binding interactions between LIR motifs on*Pf* Sec62and *Pf* Atg8 protein.

We found that the *Pf* Sec62 TSFEEL motif -*Pf* Atg8 complex was relatively more stable than the other peptides of *Pf* Sec62-*Pf* Atg8 complexes, which was attributed to its favorable van der Waals and electrostatic interactions. Even the mapping of electrostatic potentials over the surface of the peptide motifs and *Pf* Atg8 proved that the *Pf* Sec62 TSFEEL motif conferred stability due to favorable electrostatic surface complementarity. The computed interaction strength based on the non-covalent bond interactions driving the molecular recognition process was found to be higher for TSFEEL motif over other three *Pf* Sec62 motifs. Even residue-wise potential energies computed to understand the contributions of residues in complex stabilization revealed that the terminal three residues of *Pf* Sec62 TSFEEL exhibited stronger binding. Finally, analyses of multiple SMD trajectories confirmed the binding of *Pf* Sec62 TSFEEL to *Pf* Atg8 with higher affinity. Both the rupture force and PMF profiles proved to be reliable indicators for studying the relative binding affinities of the *Pf* Sec62 motifs with *Pf* Atg8. Though, the PMF profile of *Pf* Sec62 TSFEEL was similar to the free energy changes of *Pf* Sec62 QSYIDI and *Pf* Sec62 ENYDCL, the results coupled with the other analyses propose that *Pf* Sec62 might recognize and bind to *Pf* Atg8 at its L and W site through *Pf* Sec62 TSFEEL motif, whereas *Pf* Sec62 SMYKSI showed poor binding interactions in terms of potential energy, electrostatic surface complementarity, interaction strength and dissociation free energy change. On the other hand, the other two motifs of *Pf* Sec62—QSYIDI and ENYDCL exhibited moderate and variable values. Though, our computational analyses needs to be strengthened by experimental studies in near future, this study might open avenues to propose new targets for antimalarial drug discovery based on the Sec62 mediated recovER-phagy involving *Pf* Sec62-AIM motif interactions with the *Pf* Atg8 protein.

## Data Availability Statement

The raw data supporting the conclusions of this manuscript will be made available by the authors, without undue reservation, to any qualified researcher.

## Author's Note

ER-phagy is a selective autophagy induced during stress to regulate the turnover of endoplasmic reticulum, maintain its size and function to prevent excessive expansion. On the other hand, recovER-phagy ensures reversal of expanded ER to its physiological size during recovery from stress. A repertoire of autophagy regulated proteins are known to mediate selective autophagy and very recently in humans, ER translocon component—Sec62 was identified to mediate recovER-phagy during Unfolded Protein Response (UPR) by interacting with LC3 protein through its AIM/LIR motif, thus acting as an autophagy receptor. Since, *Plasmodium falciparum* is more prone to stress due to drastic transformation during their complicated life cycle and has Sec62 in the ER membrane, we investigated whether recovER-phagy takes place in this parasite by interactions of Sec62 with Atg8, a homolog of human LC3. Toward this, we analyzed peptide-protein interactions through computational studies to identify *Pf* Sec62-AIM interactions with *Pf* Atg8 to understand the molecular and structural basis of recovER-phagy in *P. falciparum*, which might hint a new drug target for developing antimalarials.

## Author Contributions

NS and AR conceptualized the study. AM carried out the computational analysis. NS and AM wrote the manuscript.

### Conflict of Interest

The authors declare that the research was conducted in the absence of any commercial or financial relationships that could be construed as a potential conflict of interest.

## Supplementary Material

Figure S1 Super-positioning of *Pf*Atg8-NDWLLP peptide complexes derived from (i) x-ray crystallography (green); (ii) GalaxyPepDock predicted peptide-protein complex (orange), and (iii) a stable conformation obtained from molecular dynamics simulations (blue).

Figure S2 RMSDs computed for the backbone atoms of the various AIM/LIR—*Pf*Atg8/*Hs*LC3 complexes, as a function of time. The RMSDs computed over the entire 50 ns MD trajectory were indicated in red color, whereas the RMSDs calculated for the trajectory of conformations obtained from dihedral PCA are indicated in black color.

Figure S3 Pull distance computed for the AIM/LIR—*Pf*Atg8/*Hs*LC3 complexes, as a function of time at a constant velocity of **(A)** 0.010 nm/ns and **(B)** 0.025 nm/ns.

Table S1 List of identified AIM/LIR motifs in *Pf*Sec62 translocon.

Table S2 Reweighted score of the predicted docked models based on different scoring functions to detect top-scoring models.

Table S3 Percentage occurrence of hydrogen bonds computed for the structurally stable conformations of AIM/LIR - Atg8/LC3 complexes.

Table S4 Residue-wise potential energies computed for the AIM/LIR motifs upon interaction with autophagy proteins.

Table S5 Residue-wise potential energies computed for the binding site residues of autophagy proteins in interaction with the various LIR motifs.

Table S6 Change in free energies of binding and dissociation of Sec62-Atg8/LC3 peptide-protein complexes.

Table S7 Excel work sheet presenting the non-covalent bond interactions observed in various peptide-protein complexes.
