# Supplementary figures and images for: Structural Analysis of PfSec62-Autophagy Interacting Motifs (AIM) and PfAtg8 Interactions for Its Implications in RecovER-phagy in Plasmodium falciparum
Source: Front Bioeng Biotechnol. 2019 Sep 25;7:240. doi: 10.3389/fbioe.2019.00240 (PMC6773812; doi:10.3389/fbioe.2019.00240)

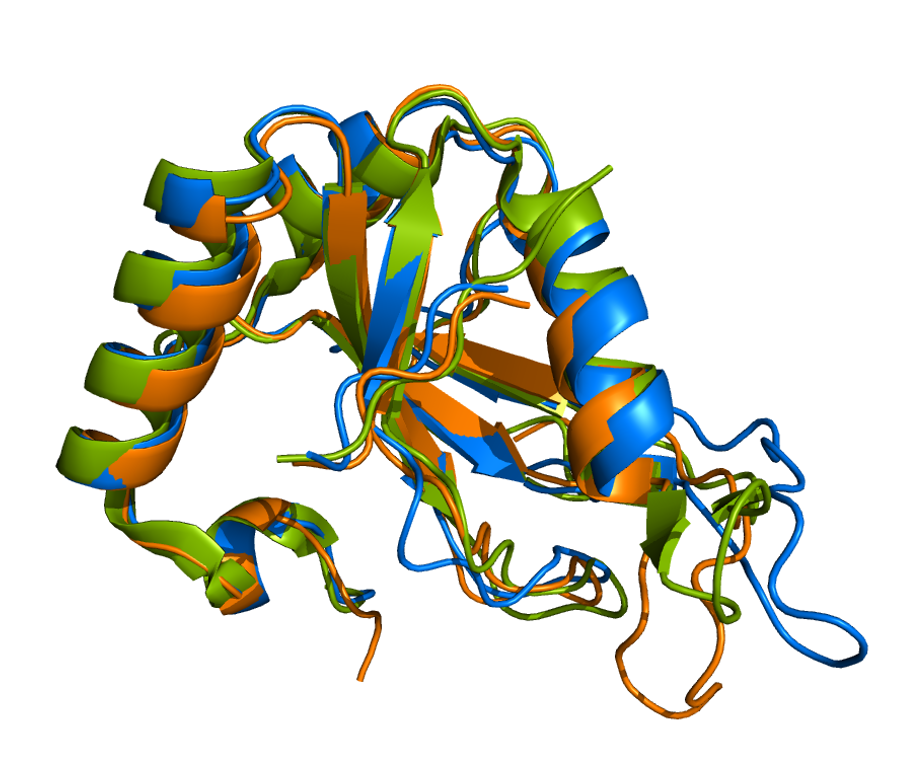

Supplement: Figure S1 — Super-positioning of PfAtg8-NDWLLP peptide complexes derived from (i) x-ray crystallography (green); (ii) GalaxyPepDock predicted peptide-protein complex (orange), and (iii) a stable conformation obtained from molecular dynamics simulations (blue). [file Image_1.TIF]

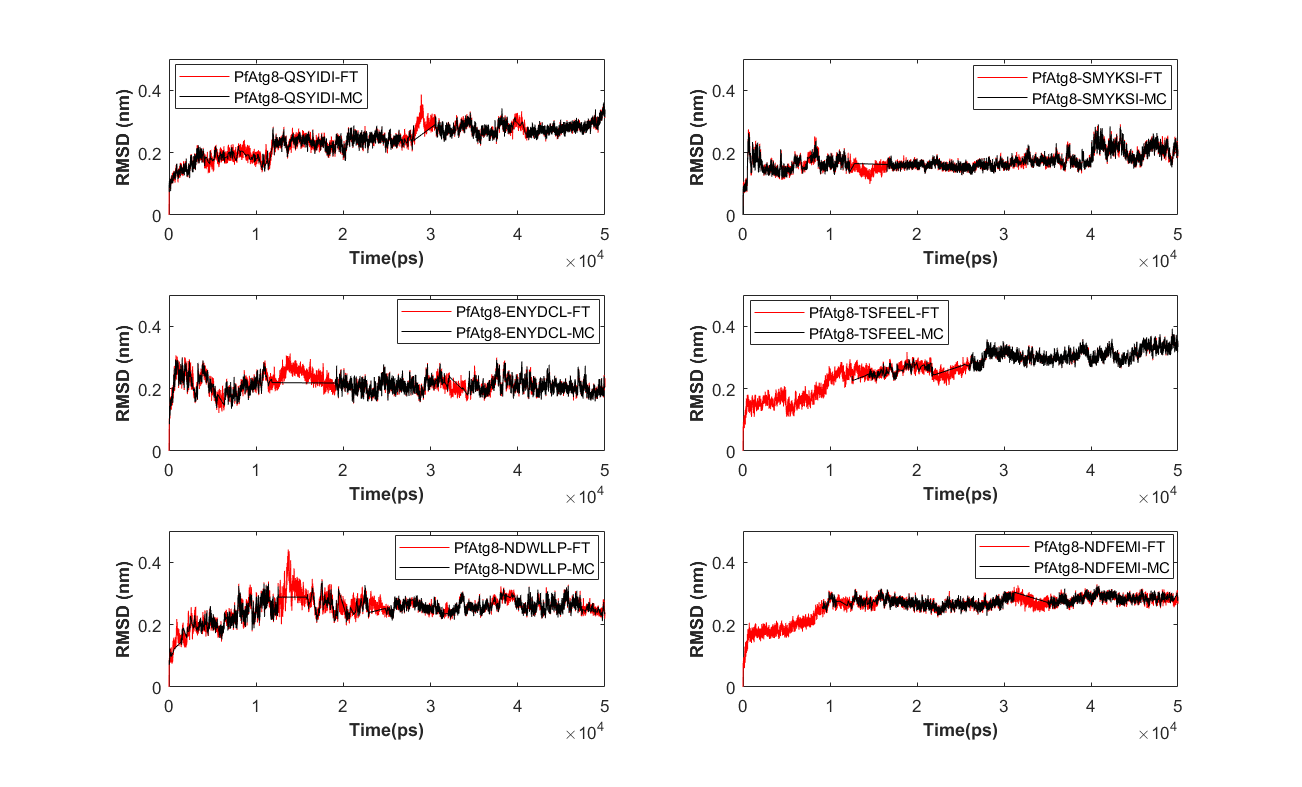

Supplement: Figure S2 — RMSDs computed for the backbone atoms of the various AIM/LIR—PfAtg8/HsLC3 complexes, as a function of time. The RMSDs computed over the entire 50 ns MD trajectory were indicated in red color, whereas the RMSDs calculated for the trajectory of conformations obtained from dihedral PCA are indicated in black color. [file Image_2.TIF]

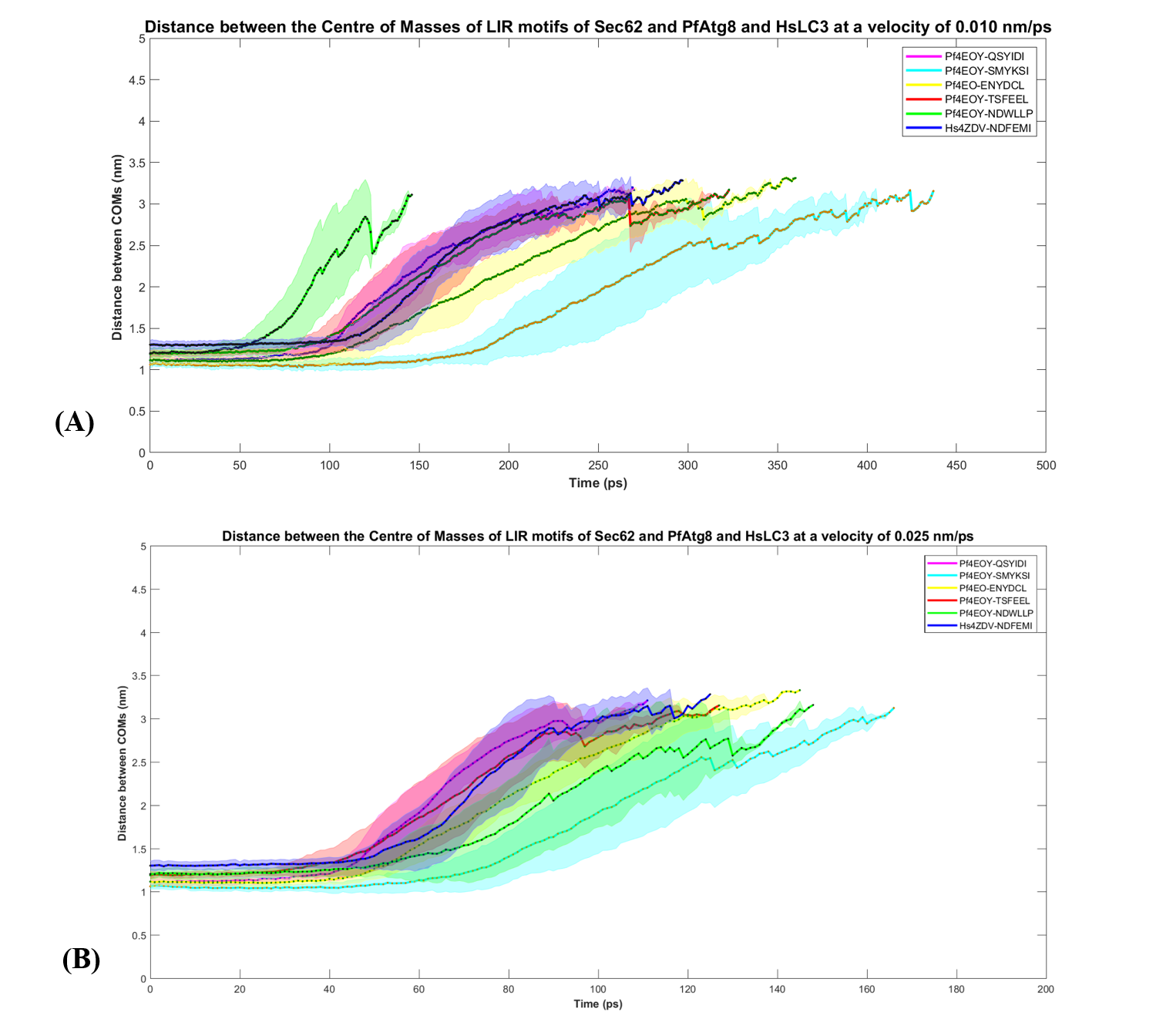

Supplement: Figure S3 — Pull distance computed for the AIM/LIR—PfAtg8/HsLC3 complexes, as a function of time at a constant velocity of (A) 0.010 nm/ns and (B) 0.025 nm/ns. [file Image_3.TIF]
