# Supplementary material for: Structural Analysis of PfSec62-Autophagy Interacting Motifs (AIM) and PfAtg8 Interactions for Its Implications in RecovER-phagy in Plasmodium falciparum
Source: Front Bioeng Biotechnol. 2019 Sep 25;7:240. doi: 10.3389/fbioe.2019.00240 (PMC6773812; doi:10.3389/fbioe.2019.00240)
Supplement: Table S1 — List of identified AIM/LIR motifs in PfSec62 translocon. [file Table_1.DOCX]

**Table S1: List of identified AIM motif sequences in *Pf*Sec62 translocon**

| **S.No** | **Motif** | **Start** | **End** | **Pattern** | **PSSM** |
| --- | --- | --- | --- | --- | --- |
| 1 | WxxL | 267 | 272 | QSYIDI | 10 |
| 2 | WxxL | 288 | 293 | SMYKSI | 10 |
| 3 | WxxL | 315 | 320 | ENYDCL | 10 |
| 4 | WxxL | 326 | 331 | TSFEEL | 11 |
