# Supplementary material for: Structural Analysis of PfSec62-Autophagy Interacting Motifs (AIM) and PfAtg8 Interactions for Its Implications in RecovER-phagy in Plasmodium falciparum
Source: Front Bioeng Biotechnol. 2019 Sep 25;7:240. doi: 10.3389/fbioe.2019.00240 (PMC6773812; doi:10.3389/fbioe.2019.00240)
Supplement: Table S2 — Reweighted score of the predicted docked models based on different scoring functions to detect top-scoring models. [file Table_2.DOCX]

**Table S2: Reweighted score of the predicted docked models based on different scoring functions to detect the best docked conformation**

| **Autophagy proteins** | ***Pf*Atg8** | | | | | | | | | | ***Hs*LC3** | |
| --- | --- | --- | --- | --- | --- | --- | --- | --- | --- | --- | --- | --- |
| **AIM/LIR Peptides** | **QSYIDI** | | **SMYKSI** | | **ENYDCL** | | **TSFEEL** | | **NDWLLP** | | **NDFEMI** | |
| **Models** | **FlexPepDock** | **FireDock** | **FlexPepDock** | **FireDock** | **FlexPepDock** | **FireDock** | **FlexPepDock** | **FireDock** | **FlexPepDock** | **FireDock** | **FlexPepDock** | **FireDock** |
| **1** | -215.524 | -81.84 | -223.193 | -51.45 | -233.435 | -77.81 | -223.235 | -78.54 | -229.807 | -73.62 | -220.447 | -95.67 |
| **2** | -206.513 | -79.93 | -221.386 | -48.61 | -233.23 | -64.32 | -221.632 | -77.1 | -226.359 | -73.08 | -215.326 | -81.81 |
| **3** | -203.831 | -77.42 | -219.988 | -40.77 | -232.923 | -60.62 | -220.581 | -72.7 | -224.848 | -67.56 | -214.413 | -74.63 |
| **4** | -197.949 | -76.53 | -217.662 | -39.89 | -232.789 | -59.7 | -219.253 | -67.77 | -217.593 | -61.28 | -213.937 | -74.34 |
| **5** | -194.272 | -75.67 | -216.38 | -37.77 | -231.694 | -54.33 | -218.642 | -66.07 | -217.465 | -60.54 | -213.586 | -73.19 |
| **6** | -194.042 | -74.05 | -216.155 | -33.2 | -231.303 | -52.09 | -218.291 | -65.56 | -216.899 | -60.06 | -211.513 | -71.99 |
| **7** | -186.579 | -73.15 | -215.886 | -29.06 | -231.086 | -51.38 | -218.133 | -64.97 | -214.994 | -59.89 | -203.745 | -71.43 |
| **8** | -173.937 | -72.66 | -215.009 | -24.18 | -231.074 | -48.64 | -217.542 | -60.8 | -212.376 | -57.61 | -202.241 | -70.22 |
| **9** | -173.235 | -66.76 | -214.59 | -18.83 | -230.813 | -47.28 | -216.585 | -59.34 | -211.865 | -55.32 | -196.734 | -68.84 |
| **10** | -166.248 | -64.24 | -191.016 | -16.75 | -230.621 | -43.43 | -208.952 | -52.51 | -163.902 | -51.21 | -194.732 | -68.42 |
