# Supplementary material for: Structural Analysis of PfSec62-Autophagy Interacting Motifs (AIM) and PfAtg8 Interactions for Its Implications in RecovER-phagy in Plasmodium falciparum
Source: Front Bioeng Biotechnol. 2019 Sep 25;7:240. doi: 10.3389/fbioe.2019.00240 (PMC6773812; doi:10.3389/fbioe.2019.00240)
Supplement: Table S3 — Percentage occurrence of hydrogen bonds computed for the structurally stable conformations of AIM/LIR - Atg8/LC3 complexes. [file Table_3.DOCX]

**Table S3: Percentage occurrence of hydrogen bonds computed for the structurally stable conformations of AIM/LIR - Atg8/LC3 complexes**

| **Complexes** | **Atoms in hydrogen bonding interactions** | | **Percentage existence** |
| --- | --- | --- | --- |
|  | **Atg8/LC3** | **AIM/LIR** |  |
| *Pf*Sec62 QSYIDI - *Pf*Atg8 | GLU17(OE2) | TYR269(OH) | 26.041 |
|  | GLU17(OE1) | TYR269(OH) | 25.013 |
|  | GLU17(O) | TYR269(OH) | 6.636 |
|  | TYR25(OH) | ASP271(OD1) | 3.291 |
|  | ARG28(NH1) | ASP271(OD1) | 7.516 |
|  | ARG28(NH1) | ASP271(OD2) | 26.417 |
|  | ARG28(NH1) | ILE272(N) | 1.773 |
|  | ARG28(NH1) | ILE272(O1) | 12.413 |
|  | ARG28(NH1) | ILE272(O2) | 68.109 |
|  | ARG28(NH2) | ASP271(OD1) | 9.518 |
|  | ARG28(NH2) | ASP271(OD2) | 5.911 |
|  | ARG28(NH2) | ILE272(O2) | 15.751 |
|  | ARG28(NH2) | ILE272(N) | 1.417 |
|  | GLU45(OE1) | GLN267(N) | 3.546 |
|  | LYS46(NZ) | GLN267(OE1) | 1.404 |
|  | LYS47(N) | SER268(O) | 52.808 |
|  | LYS48(N) | SER268(O) | 68.915 |
|  | LYS48(NZ) | SER268(OG) | 58.08 |
|  | LYS48(NZ) | TYR269(OH) | 1.713 |
|  | LYS48(O) | ILE270(N) | 87.796 |
|  | LEU50(N) | ILE270(O) | 86.257 |
|  | LEU50(O) | ILE272(N) | 8.557 |
|  | TYR113(OH) | TYR269(OH) | 27.042 |
| *Pf*Sec62 SMYKSI – *Pf*Atg8 | GLU17(OE1) | TYR290(OH) | 37.097 |
|  | GLU17(OE2) | TYR290 (OH) | 45.615 |
|  | GLU17(O) | TYR290 (OH) | 2.216 |
|  | ARG28(NH1) | ILE293(N) | 1.234 |
|  | ARG28(NE) | ILE293(O1) | 29.606 |
|  | ARG28(NH1) | SER292(OG) | 13.372 |
|  | ARG28(NH1) | ILE293(O1) | 17.171 |
|  | ARG28(NH1) | ILE293(O2) | 27.052 |
|  | ARG28(NH2) | SER292(OG) | 2.054 |
|  | ARG28(NH2) | ILE293(O1) | 16.166 |
|  | ARG28(NH2) | ILE293(O2) | 31.132 |
|  | GLU45(OE1) | SER288(N) | 2.306 |
|  | GLU45(OE2) | SER288(N) | 4.742 |
|  | GLU45(OE1) | SER288(OG) | 2.239 |
|  | GLU45(OE2) | SER288(OG) | 3.137 |
|  | LYS46(NZ) | SER288(OG) | 2.581 |
|  | LYS46(NZ) | SER288(O) | 5.869 |
|  | LYS47(N) | MET289(O) | 8.765 |
|  | LYS48(N) | MET289(O) | 43.011 |
|  | LYS48(O) | LYS291(N) | 88.295 |
|  | LEU50(O) | SER292(OG) | 10.527 |
|  | LEU50(O) | ILE292(N) | 29.443 |
|  | LEU50(N) | LYS291(O) | 87.352 |
|  | HIS67(ND1) | LYS291(NZ) | 9.545 |
|  | TYR113(OH) | TYR290(OH) | 6.616 |
| *Pf*Sec62 ENYDCL - *Pf*Atg8 | GLU17(OE1) | TYR317(OH) | 21.7 |
|  | GLU17(OE2) | TYR317(OH) | 19.711 |
|  | LYS20(NZ) | GLU315(OE2 | 1.193 |
|  | TYR25(OH) | TYR317(OH) | 1.071 |
|  | ARG28(NE) | CYS319(O) | 1.167 |
|  | ARG28(NH1) | CYS319(O) | 10.789 |
|  | ARG28(NH2) | CYS319(O) | 42.656 |
|  | GLU45(OE1) | GLU315(N) | 11.514 |
|  | GLU45(OE2) | GLU315(N) | 11.232 |
|  | GLU45(OE1) | ASN316(ND2) | 1.847 |
|  | GLU45(OE2) | ASN316(ND2) | 1.302 |
|  | GLU45(O) | ASN316(ND2) | 6.132 |
|  | LYS46(NZ) | ASN316(OD1) | 1.572 |
|  | LYS46(NZ) | TYR317(O) | 1.552 |
|  | LYS46(NZ) | ASP318(OD1) | 42.316 |
|  | LYS46(NZ) | ASP318(OD2) | 35.459 |
|  | LYS47(N)Z | GLU315(OE1) | 5.471 |
|  | LYS47(N)Z | GLU315OE2 | 6.177 |
|  | LYS48(NZ) | GLU315(OE1) | 41.918 |
|  | LYS48(NZ) | GLU315OE2 | 46.517 |
|  | LYS48(O) | ASP318(N) | 50.487 |
|  | LEU50(N) | ASP318(O) | 97.768 |
| *Pf*Sec62 TSFEEL - *Pf*Atg8 | GLU17(OE1) | THR326(N) | 1.489 |
|  | GLU17(OE2) | THR326(N) | 1.907 |
|  | GLU17(OE1) | SER327(N) | 3.285 |
|  | GLU17(OE1) | SER327(OG) | 6.521 |
|  | GLU17(OE2) | SER327(OG) | 4.233 |
|  | LYS24(NZ) | THR326(OG)1 | 2.535 |
|  | TYR25(OH) | GLU330(OE1) | 8.527 |
|  | TYR25(OH) | GLU330OE2 | 6.288 |
|  | ARG28(NH1) | GLU330(OE1) | 26.886 |
|  | ARG28(NH1) | GLU330OE2 | 26.972 |
|  | ARG28(NH2) | GLU330(OE1) | 35.548 |
|  | ARG28(NH2) | GLU330OE2 | 27.058 |
|  | ARG28(NH2) | LEU331(N) | 4.663 |
|  | ARG28(NH2) | LEU331(O1) | 33.739 |
|  | ARG28(NH2) | LEU331(O2) | 31.697 |
|  | LYS46(NZ) | GLU329(OE1) | 44.777 |
|  | LYS46(NZ) | GLU329OE2 | 42.107 |
|  | LYS48(NZ) | SER327(OG) | 36.852 |
|  | LYS48(O) | GLU329(N) | 97.145 |
|  | LEU50(O) | LEU331(N) | 22.874 |
|  | LEU50(N) | GLU329(O) | 92.568 |
|  | HIS67NE2 | GLU329(OE1) | 1.772 |
| *Pf*Atg3-NDWLLP - *Pf*Atg8 | GLU17(OE1) | TRP105NE1 | 56.876 |
|  | GLU17(OE2) | TRP105NE1 | 42.431 |
|  | ARG28(NH1) | PRO108(O1) | 16.989 |
|  | ARG28(NH1) | PRO108(O2) | 30.749 |
|  | ARG28(NH2) | PRO108(O2) | 2.027 |
|  | LYS46(NZ) | ASN103(OD1) | 4.945 |
|  | LYS46(NZ) | ASN103(O) | 12.161 |
|  | LYS46(NZ) | ASP104(OD1) | 4.481 |
|  | LYS46(NZ) | ASP104(OD2) | 6.029 |
|  | LYS48(NZ) | ASP104(OD1) | 54.776 |
|  | LYS48(NZ) | ASP104(OD2) | 58.535 |
|  | LYS48(O) | LEU106(N) | 98.297 |
|  | LEU50(N) | LEU106(O) | 99.189 |
| *Hs*Sec62 NDFEMI - *Hs*LC3 | GLY1(N) | ASP362(OD1) | 2.049 |
|  | GLY1(N) | ASP362(OD2) | 1.07 |
|  | GLN26(NE2) | ASN361(OD1) | 1.007 |
|  | GLN26(NE2) | ASN361(ND2) | 2.004 |
|  | GLN26(OE1) | ASN361(ND2) | 24.845 |
|  | LYS30(NZ) | ILE366(O1) | 70.103 |
|  | LYS30(NZ) | ILE366(O2) | 22.625 |
|  | ASP48(OD1) | ASN361(N) | 5.627 |
|  | ASP48(OD2) | ASN361(N) | 3.353 |
|  | ASP48(OD1) | ASN361(ND2) | 1.007 |
|  | ASP48(OD2) | ASN361(ND2) | 1.151 |
|  | LYS49(NZ) | ASN361(OD1) | 6.804 |
|  | LYS49(NZ) | ASN361(O) | 6.778 |
|  | LYS49(NZ) | PHE363(O) | 2.238 |
|  | LYS49(NZ) | GLU364(OE1) | 49.699 |
|  | LYS49(NZ) | GLU364OE2 | 42.661 |
|  | THR50(N) | ASP362(O) | 2.085 |
|  | LYS51(N) | ASP362(O) | 4.027 |
|  | LYS51(N)Z | ASP362(OD1) | 36.629 |
|  | LYS51(N)Z | ASP362(OD2) | 69.627 |
|  | LYS51(O) | GLU364(N) | 95.82 |
|  | LEU53(O) | ILE366(N) | 65.088 |
|  | LEU53(N) | GLU364(O) | 95.101 |
|  | ARG70(NH1) | GLU364(OE1) | 41.78 |
|  | ARG70(NH1) | GLU364OE2 | 46.193 |
|  | ARG70(NH2) | GLU364(OE1) | 24.764 |
|  | ARG70(NH2) | GLU364OE2 | 25.789 |
