# Supplementary material for: Structural Analysis of PfSec62-Autophagy Interacting Motifs (AIM) and PfAtg8 Interactions for Its Implications in RecovER-phagy in Plasmodium falciparum
Source: Front Bioeng Biotechnol. 2019 Sep 25;7:240. doi: 10.3389/fbioe.2019.00240 (PMC6773812; doi:10.3389/fbioe.2019.00240)
Supplement: Table S4 — Residue-wise potential energies computed for the AIM/LIR motifs upon interaction with autophagy proteins. [file Table_4.DOCX]

**Table S4:** **Residue-wise potential energies computed for the AIM/LIR motifs upon interaction with autophagy receptors**

| **Residue Position in the AIM/LIR motif** | ***Pf*Atg8** | | | | | ***Hs*LC3** |
| --- | --- | --- | --- | --- | --- | --- |
|  | **QSYIDI** | **SMYKSI** | **ENYDCL** | **TSFEEL** | **NDWLLP** | **NDFEMI** |
| **1** | 69.95 | 88.09 | -66.29 | 78.81 | 102.16 | 83.19 |
| **2** | -32.02 | -30.03 | -23.54 | -19.84 | -155.42 | -196.42 |
| **3** | -47.11 | -49.25 | -27.96 | -41.63 | -48.79 | -40.36 |
| **4** | -32.46 | 66.20 | -169.13 | -175.24 | -33.26 | -252.76 |
| **5** | -132.46 | -9.20 | -20.12 | -159.13 | -12.26 | -23.75 |
| **6** | -147.49 | -154.96 | -119.36 | -137.39 | -130.92 | -175.54 |
