# Supplementary material for: Structural Analysis of PfSec62-Autophagy Interacting Motifs (AIM) and PfAtg8 Interactions for Its Implications in RecovER-phagy in Plasmodium falciparum
Source: Front Bioeng Biotechnol. 2019 Sep 25;7:240. doi: 10.3389/fbioe.2019.00240 (PMC6773812; doi:10.3389/fbioe.2019.00240)
Supplement: Table S5 — Residue-wise potential energies computed for the binding site residues of autophagy proteins in interaction with the various LIR motifs. [file Table_5.DOCX]

**Table S5: Residue-wise potential energies computed for the binding site residues of autophagy receptors in interaction with the various AIM/LIR motifs**

| **Autophagy proteins** | **Binding site** | **Residues** | **AIM motifs** | | | | | **Autophagy receptor proteins** | **Binding site** | **Residues** | **LIR motifs** |
| --- | --- | --- | --- | --- | --- | --- | --- | --- | --- | --- | --- |
|  |  |  | **QSYIDI** | **SMYKSI** | **ENYDCL** | **TSFEEL** | **NDWLLP** |  |  |  | **NDFEMI** |
| ***Pf*Atg8** | **W-site** | \| Glu17 \| \| --- \| | **0.65** | **-46.61** | **56.6** | **16.33** | **34.21** | ***Hs*LC3** | **W-site** | \| Glu19 \| \| --- \| | **65.86** |
|  |  | \| Ile21 \| \| --- \| | -1.84 | **-2.77** | **-1.71** | **-1.98** | -8.55 |  |  | Ile23 | **-4.73** |
|  |  | Tyr25 | -2.08 | -1.8 | -0.95 | -2.46 | **-5.39** |  |  | Lys30 | **-139.89** |
|  |  | Arg28 | **-137.12** | **-40.42** | **-101.58** | **-171.2** | **-73.38** |  |  | Pro32 | -2.64 |
|  |  | Pro30 | -2.55 | -1.94 | -2.32 | -4.9 | -1.77 |  |  | Ile34 | **-1.97** |
|  |  | Lys48 | **-43.41** | **11.1** | **-112.81** | **-67.18** | **-112.84** |  |  | Lys51 | **-145.07** |
|  |  | Leu50 | **-12.05** | **-16.5** | **-13.68** | **-14.67** | **-17.38** |  |  | Leu53 | **-16.11** |
|  |  | Tyr113 | **-3.24** | **-0.55** | -1.28 | -3.47 | -0.63 |  |  | Phe108 | -2.71 |
|  | **L-Site** | \| Phe49 \| \| --- \| | **-19.03** | **-19.24** | **-18.92** | **-23.92** | **-18.43** |  | **L-Site** | \| Ile35 \| \| --- \| | **-16.62** |
|  |  | Leu50 | **-12.05** | **-16.05** | **-13.68** | **-14.67** | **-17.38** |  |  | Phe52 | **-7.45** |
|  |  | Val51 | **-5.34** | **-5.55** | **-7.61** | **-5.27** | -5.45 |  |  | Val54 | **-6.34** |
|  |  | Pro52 | **-5.25** | **-4.74** | -3.33 | **-4.51** | -3.29 |  |  | Pro55 | **-3.42** |
|  |  | Met55 | **-3.77** | **-2.79** | **-3.52** | -3.48 | -2.16 |  |  | Val58 | **-0.18** |
|  |  | Phe60 | -1.28 | -3.54 | -1.01 | **-1.02** | -1.5 |  |  | Leu63 | **-3.99** |
|  |  | Ile63 | **-4.59** | **-5.94** | **-4.61** | **-4.2** | **-4.55** |  |  | Ile66 | -0.03 |
|  |  | His67 | -3.58 | **-10.26** | **-1.99** | **-2.4** | -1.87 |  |  | Ile67 | **-131.2** |
|  |  |  |  |  |  |  |  |  |  | Arg70 | **-16.62** |

***Note:*** Values represented in bold are AIM/LIR motifs participating in non-covalent bond interactions
