# Supplementary material for: Structural Analysis of PfSec62-Autophagy Interacting Motifs (AIM) and PfAtg8 Interactions for Its Implications in RecovER-phagy in Plasmodium falciparum
Source: Front Bioeng Biotechnol. 2019 Sep 25;7:240. doi: 10.3389/fbioe.2019.00240 (PMC6773812; doi:10.3389/fbioe.2019.00240)
Supplement: Table S6 — Change in free energies of binding and dissociation of Sec62-Atg8/LC3 peptide-protein complexes. [file Table_6.DOCX]

**Table S6: Change in free energies of binding and dissociation of Sec62-Atg8/LC3 peptide-protein complexes**

| **S.No** | **Peptide-Protein complex** | **mmPBSA method** | **Pull velocity = 0.010 nm/ns** | | **Pull velocity = 0.025 nm/ns** | |
| --- | --- | --- | --- | --- | --- | --- |
|  |  | **ΔG_binding_ (kJ/mol)** | **ΔG_dissociation_ (kJ/mol)** | **Dissociation constant (K_d_)** | **ΔG_dissociation_ (kJ/mol)** | **Dissociation constant (K_d_)** |
| 1 | *Pf*QSYIDI - *Pf*Atg8 | -3.76 x 10^8^ | 341.2285 | 3.17 x 10^-58^ | 1.19 | 0.62 |
| 2 | *Pf*SMYKSI - *Pf*Atg8 | -8.15 x 10^5^ | 309.68 | 6.56 x 10^-53^ | 1.69 | 0.51 |
| 3 | *Pf*ENYDCL - *Pf*Atg8 | -4.29 x 10^6^ | 297.4947 | 7.42 x 10^-51^ | 1.48 | 0.56 |
| 4 | *Pf*TSFEEL - *Pf*Atg8 | -6.29 x 10^6^ | 337.9418 | 1.13 x 10^-57^ | 1.22 | 0.62 |
| 5 | *Pf*NDWLLP - *Pf*Atg8 | -1.73 x 10^6^ | 471.6232 | 3.38 x 10^-80^ | 1.36 | 0.58 |
| 6 | *Hs*NDFEMI - *Hs*LC3 | -8.97 x 10^5^ | 434.3202 | 6.52 x 10^-74^ | 1.54 | 0.54 |

* **ΔG_binding_ is computed using mmPBSA method and ΔG_dissociation_ is calculated using Constant velocity steered MD simulations**
